# Mitochondrial Ndufa4l2 Enhances Deposition of Lipids and Expression of Ca9 in the TRACK Model of Early Clear Cell Renal Cell Carcinoma

**DOI:** 10.3389/fonc.2021.783856

**Published:** 2021-12-14

**Authors:** Kristian B. Laursen, Qiuying Chen, Francesca Khani, Nabeel Attarwala, Steve S. Gross, Lukas Dow, David M. Nanus, Lorraine J. Gudas

**Affiliations:** ^1^ Department of Pharmacology, New York Presbyterian Hospital, Weill Cornell Medicine, New York, NY, United States; ^2^ Department of Pathology and Laboratory Medicine, New York Presbyterian Hospital, Weill Cornell Medicine, New York, NY, United States; ^3^ Department of Urology, New York Presbyterian Hospital, Weill Cornell Medicine, New York, NY, United States; ^4^ Department of Medicine, New York Presbyterian Hospital, Weill Cornell Medicine, New York, NY, United States; ^5^ Department of Biochemistry, New York Presbyterian Hospital, Weill Cornell Medicine, New York, NY, United States; ^6^ Graduate School of Medical Sciences, New York Presbyterian Hospital, Weill Cornell Medicine, New York, NY, United States; ^7^ Division of Hematology and Medical Oncology, Department of Medicine, New York Presbyterian Hospital, Weill Cornell Medicine, New York, NY, United States

**Keywords:** clear cell renal cell carcinoma, hypoxia, HIF1, von Hippel-Lindau, proximal tubules, lipids, mitochondria

## Abstract

Mitochondrial dysfunction and aberrant glycolysis are hallmarks of human clear cell renal cell carcinoma (ccRCC). Whereas glycolysis is thoroughly studied, little is known about the mitochondrial contribution to the pathology of ccRCC. Mitochondrial Ndufa4l2 is predictive of poor survival of ccRCC patients, and in kidney cancer cell lines the protein supports proliferation and colony formation. Its role in ccRCC, however, remains enigmatic. We utilized our established ccRCC model, termed Transgenic Cancer of the Kidney (TRACK), to generate a novel genetically engineered mouse model in which dox-regulated expression of an shRNA decreases Ndufa4l2 levels specifically in the renal proximal tubules (PT). This targeted knockdown of Ndufa4l2 reduced the accumulation of neutral renal lipid and was associated with decreased levels of the ccRCC markers carbonic anhydrase 9 (CA9) and Enolase 1 (ENO1). These findings suggest a link between mitochondrial dysregulation (i.e. high levels of Ndufa4l2), lipid accumulation, and the expression of ccRCC markers ENO1 and CA9, and demonstrate that lipid accumulation and ccRCC development can potentially be attenuated by inhibiting Ndufa4l2.

## Introduction

Clear cell renal cell carcinoma (ccRCC) is the most common type of kidney cancer, affecting nearly 300,000 people worldwide each year (1). Histologically, the pathology is evident by “clear cell” tumors in the kidney, i.e. cell clusters of proximal tubule (PT) origin in which the accumulation of lipids and glycogen is visualized as a transparent cytosol (2, 3). The clear cell morphology is associated with a transition from mitochondrial respiration, driven by the electron transport chain (ETC), to cytoplasmic glycolysis as the major source of ATP production (4–7). This altered metabolism, referred to as the Warburg effect, is observed in many types of cancers and can potentially be exploited for treatment (6, 8, 9). Based on the glycolytic dependence of cancer cells, inhibitors of glycolysis have been suggested for cancer therapy. However, without restored mitochondrial function, inhibition of glycolysis may be insufficient to inhibit cancer cell proliferation (6), and therapies for ccRCC could potentially be improved by enhancing mitochondrial function while inhibiting glycolysis.

We have previously modeled various aspects of ccRCC using our TRACK (Transgenic Cancer of the Kidney) kidney cancer mouse model (10, 11). In this model, a constitutively active transcription factor, HIF1α (P402A, P564A, N803A), is expressed specifically in the PT, where it drives a ‘hypoxic’ transcriptional signature similar to that observed in human ccRCC (10). TRACK mice emulate early onset ccRCC in their accumulation of lipid-filled “clear” cells, distorted tubular structure, increased lipid and glycogen deposition, and multiple renal cysts (11). The observation that HIF1α activation is sufficient to drive these changes in TRACK suggests that transcriptional targets of HIF1α, potentially mitochondrial NADH dehydrogenase 1 alpha subcomplex 4-like 2 (NDUFA4L2), may contribute to the metabolic reprogramming observed in human ccRCC (10). Notably, activation of HIF2α in kidney PT cells also causes abnormal glycogen deposition, but unlike the transcriptional profile of HIF1α transgenic kidneys (TRACK), the profile of HIF2α transgenic kidneys in the TRACK mouse model does not resemble that of human ccRCC (12). Overall, the activation of HIF1α in the PTs of immunocompetent TRACK mice causes transcriptional and metabolic reprogramming which mirrors the changes associated with early development of ccRCC in patients.

The activation of HIF1 signaling is a key feature of ccRCC, and among the HIF1 upregulated target genes is Ndufa4l2 (13, 14). This nuclear gene encodes a mitochondrial protein whose elevated expression level is predictive of poor clinical outcome in ccRCC patients (15, 16). Hypoxia induced transcription of NDUFA4L2 occurs through hypoxia-responsive elements in the gene’s promoter region (14). *In vivo* accumulation of HIF1α protein markedly induces Ndufa4l2 expression, whereas accumulation of HIF2α does not (12). A seminal study by Tello et al. (14) reported that depletion of HIF1α, but not HIF2α, reduces NDUFA4L2 transcript in RCC4 kidney cancer cells. Further corroborating this finding, a genomic deletion of Hif1α abolishes induction of Ndufa4l2 in MEFs (14). Consequently, these data suggest that transcription of NDUFA4L2 is dependent on activation of HIF1α rather than HIF2α.

Biologically, NDUFA4L2 plays a key role when cells reduce their oxygen consumption in response to hypoxic conditions. This was demonstrated by depletion and overexpression experiments in both MEFs and HeLa cells, and supported by biochemical assays of the ETC complexes showing that increased NDUFA4L2 expression selectively impairs the activity of Complex-I (14). The ability of NDUFA4L2 to impair ETC function argues for a role of NDUFA4L2 in the shift from oxidative phosphorylation to glycolysis. Furthermore, the observed protective role of NDUFA4L2 in hypoxia/reoxygenation during ischemia/reperfusion (17) implicates NDUFA4L2 expression as a contributor to cellular survival during hypoxic conditions. Conversely, the depletion of NDUFA4L2 impaired proliferation/survival of mouse embryonic fibroblasts, HeLa, and RCC4 cell lines (14, 15), suggesting that targeting NDUFA4L2 can provide an effective means to inhibit ccRCC cell proliferation.

Here we genetically modified our TRACK mouse model of ccRCC to express an shRNA which selectively targets Ndufa4l2 transcripts in the kidney PT. This allowed us to evaluate the effects of attenuating Ndufa4l2 expression in a mouse model of early-stage patient ccRCC. Results demonstrated that decreasing Ndufa4l2 transcript levels in the mouse kidneys evoked a marked reduction in the accumulation of neutral renal lipids and additionally decreased the level of carbonic anhydrase 9 (Car9) and α-Enolase 1 (Eno1), well-established ccRCC biomarkers. Together, our findings suggest that Ndufa4l2 may play a fundamental role as a driver of ccRCC lipid accumulation and expression of CA9 and ENO1 cancer biomarkers.

## Materials and Methods

### DNA Constructs

We generated the Ndufa4l2 knockdown constructs as follows. Chemically synthesized DNA oligomers (97-mers) with Ndufa4l2-targeting hairpins were acquired and PCR-amplified (miRE-Eco-Rev/miRE-Xho-Fw primers), and inserted into the doxycycline (dox) inducible L3GGEPPIR vector and the cTGME targeting vector using EcoRI/XhoI cloning (18). The integrity of the hairpin region of each resulting construct were confirmed by DNA sequencing. The RNA hairpins were designed using Splash and transOMIC online resources (splashrna.mskcc.org and www.transomic.com).

The LucR/LucF dual reporter construct was generated as follows. Ndufa4l2 cDNA (NM_001098789.1) was cloned from kidney RNA (TRACK mouse DNA). In brief, the cDNA was amplified using the mNdufa4l2 (–)E4/mNdufa4l2(+)X1 primer-pair (**Table S1**), and inserted into the psiCHECK2 vector (Cat# C8021, Promega, WI) immediately downstream of the LucR coding region. The resulting psiCHECK2 mNdufa4l2 constructs were confirmed by DNA sequencing.

### Transfection, Selection, and Assay of Knockdown Efficiencies of 11 Ndufa4l2-Targeting Hairpins

Generation of viral particles and transduction of F9 cells was previously described (19). F9 cells were transduced with knockdown vectors L3GGEPPIR shNdufa4l2.<ID#> or L3GGEPPIR shLucR (control). After puromycin selection each polyclonal F9 cell line was transiently transfected with the dual luciferase reporter, psiCHECK2 mNdufa4l2, using lipofectamine 2000 (Cat. #11668019, Invitrogen), and upon recovery the cells were treated with control vehicle or doxycycline (Cat. #D3447, Sigma-Aldrich) to induce coexpression of Green Fluorescent Protein (GFP) and the Ndufa4l2 shRNAs. The dox-treatment induced shRNA expression in more than 95% of the cells, as determined by visualization of GFP expression. After 24 hr. of dox-treatment, we harvested the cells, and we measured Firefly (LucF) and Renilla (LucR) luciferase activities sequentially in a luminometer using the Dual-luciferase Reporter Assay system (Cat. #E1910, Promega). For data-processing the Renilla signal was normalized to the Firefly signal.

### Generation of a Doxycycline-Inducible shNDUFA4L2 Founder Embryonic Stem Cell Line

The targeting plasmid DNA (cTGME) and Flp-recombinase expression vector (pCAGGS-FLPe) were introduced by nucleofection into the KH2 ES cell line (20). The transfected cells were seeded on DR4 feeder cells for overnight recovery, after which the media was replenished with hygromycin (18). Resulting colonies were expanded and genotyped for targeting of the *Col1a1* locus using the Col1A1(+)/Col1A1 (–) and Col1A1(+)/SAdpA (–) primer-pairs (wild-type and transgenic, respectively).

### Mating and Genotyping of Mouse Lines

The tet-responsive shNDUFA4L2 founder ES cell lines were injected into tetraploid blastocysts (C57Bl/6) to develop transgenic embryos (NYU GEMM facility, NY, NY). Three transgenic founder mice were identified, and cross-bred with two established transgenic mouse lines, the TRACK line (11), and a tet-Transactivator (tTA) driven by a 2.7 kb gamma-glutamyltransferase 1 (ggt) promoter, the ggt-tTA line (21). In the resulting triple-positive TANdu mouse line (TANdu; tTA-shNdufa4l2-TRACK) the Ndufa4l2 levels are negatively regulated by the ggt promoter, which *via* the tet-transactivator (tTA) induces expression of the shRNA. Furthermore, the induction of shNdufa4l2 can be prevented by supplementation of the drinking water with doxycycline (2mg ml^-1^). The allelic copy numbers of shNdufa4l2, TRACK, and ggt-tTA were routinely determined by quantitative real-time PCR using primer pairs TETO(+)B/EGFP (–); shNdufa4l2, hHIF1aP564A-F/hHIF1aN803A-R; TRACK, and tta507_F/tta507_R; ggt-tTA, (see **Tables S2** and **S3** for details). All evaluated animals were biallelic for each of the three transgenes. All mice included in the analysis were sacrificed at six months of age. The harvested tissue was either fixed in 4% paraformaldehyde (for immunostaining) or stored at -80°C until the time of analysis.

### Western Blotting

Immunoblotting of kidney proteins were performed as previously described (22, 23). Band intensities were quantified using ImageJ software (v1.53), and Ndufa4l2 values were each normalized to the corresponding β-actin intensities (24). Antibodies and dilutions are specified in the supplementary information.

### Staining and Imaging

Immunostaining of the mouse kidney cortex sections was performed as previously described (25). In brief, paraformaldehyde-fixed kidney sections were cut to 7 μm and mounted on glass slides by the Cornell Core Facility. Following deparaffinization in Histoclear-II (Cat. #HS-202, National Diagnostics) and unmasking by pressure cooker, boiling for 5 minutes in citrate buffer (Cat.# H-3300, Vector Labs), the sections were stained with primary antibody (specified in the supplement) in a 1:200 dilution overnight at 4°C. The staining was detected using Alexa Fluor Plus secondary antibodies (Invitrogen) in a 1:1,000 dilution overnight at 4°C. Frozen sections were fixed in paraformaldehyde (4%), followed by washing in PBS. The sections were incubated 15 minutes at room temperature in PBS with Bodipy, 1:100 (Cat.#D3861, Invitrogen), and NucBlue, 1:100 (Cat.#R37605, Invitrogen), followed by PBS washes. The sections were visualized with a Nikon TE2000 inverted microscope using NIS Elements.

### Matrix-Assisted Laser Desorption Ionization Imaging Mass Spectrometry

Kidney samples were snap-frozen in 2% carboxymethyl-cellulose (CMC, Sigma, #C5013), then cryo-sectioned at −20°C into 10 μm thickness sections that were thaw-mounted onto indium tin oxide (ITO, Bruker Daltonics) coated glass slides and stored at -80°C for the MALDI-IMS study. ITO-slides with tissue sections were transferred from -80°C to a vacuum chamber and dried for 30 min prior to matrix deposition using 9-Aminoacridine (9-AA, 5mg ml^-1^ in 70% methanol) and matrix sprayer HTX TM-Sprayer™ (HTX Technologies LLC, NC). Matrix-coated kidney sections were loaded for MALDI-IMS data acquisition on the same day of matrix deposition on a 7T Scimax-MRMS mass spectrometer (Bruker Daltonics, USA) equipped with a SmartBeam II laser and a MALDI source. Acquired image data was exported to Scils Lab software for visualization and statistical analysis (SCiLS, Bremen, Germany). Compound identifications were assigned based on both accurate mass and isotope pattern matches (<1 ppm). Mean image intensities between corresponding kidney regions were compared using Student t-tests (2-tailed).

### Data Processing

For statistical analyses data from at least three independent animals were analyzed using one-way ANOVA. The standard error of mean was determined for each of the data sets, and ANOVA values of p<0.05 among compared samples were assigned statistical significance.

## Results

### Activation of HIF1α, but Not HIF2α, Induces Ndufa4l2, Car9, Eno1, and Gpi1

**Figure 1:**
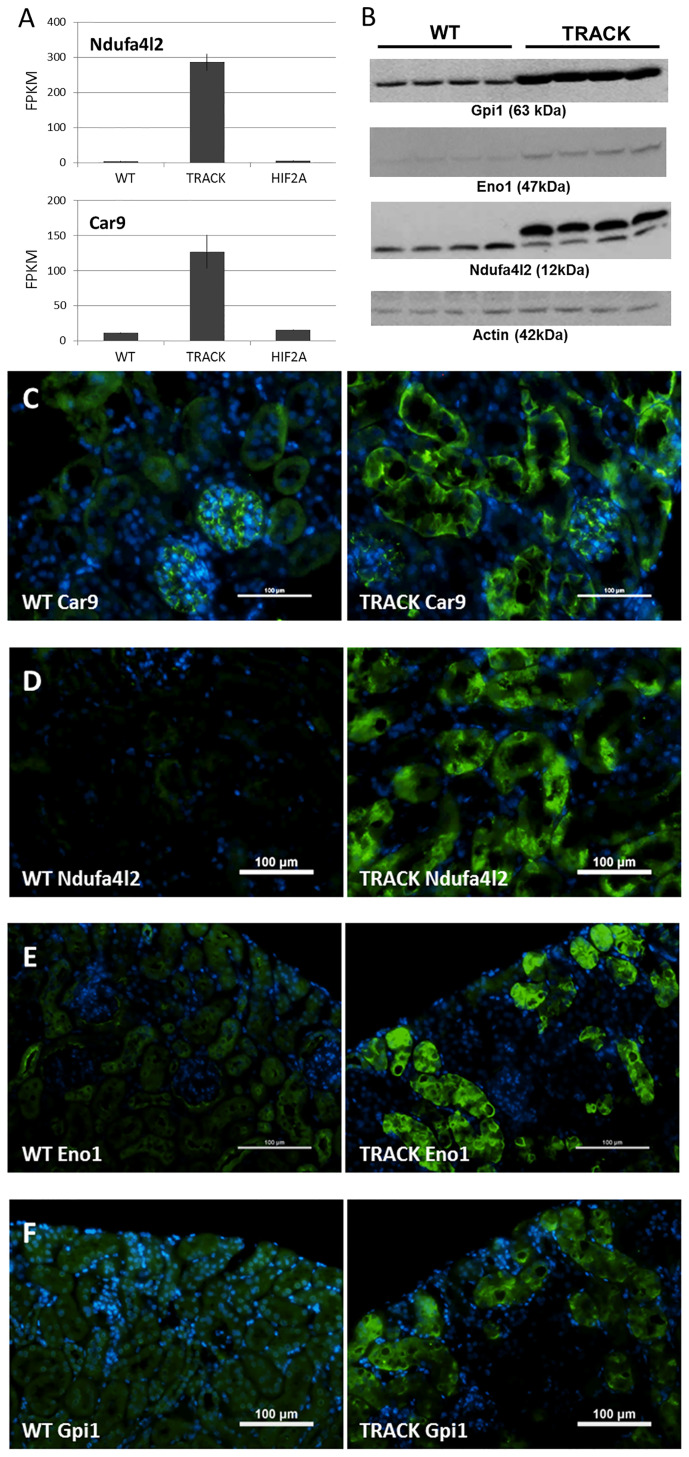
Ndufa4l2 and Car9 levels in the TRACK (HIF1α) ccRCC mouse model. **(A)** Transcript levels of Ndufa4l2 and Car9 in TRACK datasets are elevated in “TRACK” (HIF1α) relative to “Normal” (WT) samples, as previously reported (15, 28). Note that Ndufa4l2 and Car9 levels are normal in the HIF2α mouse model. **(B)** Western blotting of Ndufa4l2, Actin, Eno1, and Gpi1. The intensities of correctly sized bands were increased in the TRACK mice, thus validating the specificities of the antibodies. All proteins were detected on the same membrane. **(C–F)** Immunofluorescence of Car9, Ndufa4l2, Eno1, and Gpi1 in WT and TRACK kidneys (shown in green), DAPI nuclear staining in blue. **(C)** Car9, **(D)** Ndufa4l2, **(E)** Eno1, **(F)** Gpi1. Magnification; x200, scale bar; 100 μm. Representative images are shown. Note that under denaturing conditions **(B)**, the Ndufa4l2 antibody recognizes also Ndufa4, however, the absence of signal in WT kidneys **(C)** confirms the specificity in the immunofluorescence detection.

Carbonic Anhydrase 9 (human; CA9, mouse; Car9) is one of the most commonly used markers of ccRCC, and elevated expression is an independent adverse predictor of survival (26, 27). Similarly, high levels of NDUFA4L2 are associated with an unfavorable prognosis in ccRCC patients (15, 28). As previously reported, elevated Ndufa4l2 and Carbonic Anhydrase 9 (Car9) transcript levels are recapitulated in our TRACK (HIF1α) kidney cancer mouse model (11) relative to normal kidney (WT) (15, 28). In contrast, Ndufa4l2 and Car9 transcript levels are not elevated in our HIF2α overexpressing mouse model (**Figure 1A**).

In addition to Ndufa4l2, we performed immunoblotting of α-Enolase 1 (Eno1), a prognostic marker in clear cell renal cell carcinoma (29), and glucose-6-phosphate isomerase (Gpi1), independent adverse prognostic markers of ccRCC aggressiveness and progression-free survival (30). We found increased Ndufa4l2, Eno1, and Gpi1 protein levels in the TRACK kidney cortices relative to WT (**Figure 1B**), thus confirming elevated levels of these ccRCC biomarkers in the TRACK mouse model. We next immunostained mouse kidneys (WT and TRACK) for Car9, Ndufa4l2, Eno1, and Gpi1 (**Figures 1C** **–F**), and detected staining patterns for these proteins that matched the tubular clear cell pathology in the TRACK kidneys. Thus, several markers of human ccRCC pathology display clear-cell associated staining patterns in the TRACK model. We evaluated testis, pancreas, and liver of adult TRACK males for Ndufa4l2 expression (**Figure S1**), and found no detectable protein in these tissues.

### *In Vivo* Targeting of Ndufa4l2 Using shRNA Expression

**Figure 2:**
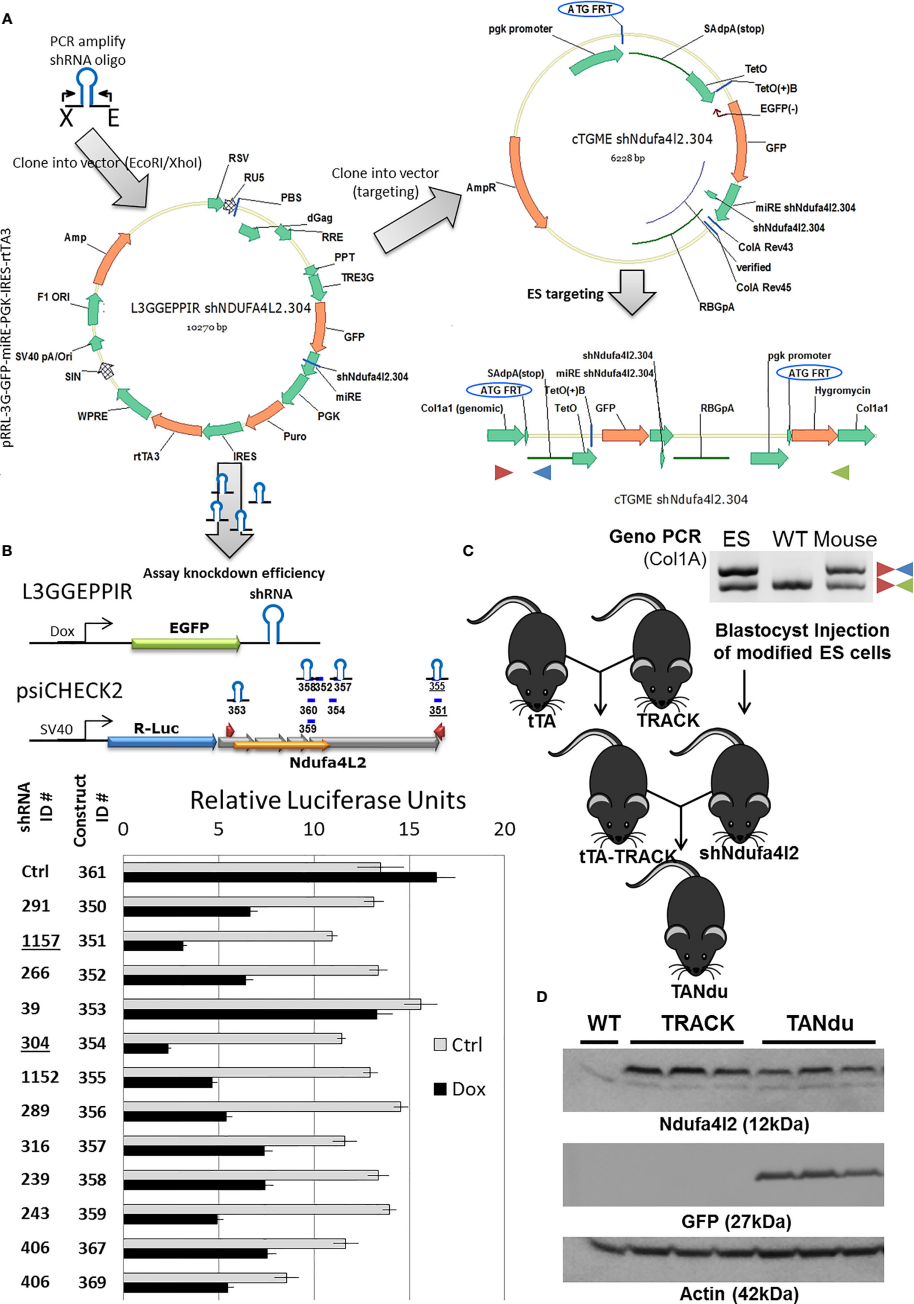
Strategy for the generation of an inducible Ndufa4l2 knockdown ES cell line. **(A)** Several shRNA sequences were cloned into the L3GGEPPIR vector, and evaluated in gene-silencing assays using a luciferase reporter system. The most potent hairpin (shNdufa4l2.304) was cloned into the cTGME vector for genomic targeting into the modified Col1a1 locus of the KH2 ES cell line. **(B)** Ndufa4l2 gene silencing assessed in polyclonal cell lines with specific shRNA expression. The knockdown potency of individual shRNAs was assessed by transient introduction of an Ndufa4l2-tagged psiCHECK2 dual luciferase reporter. In brief, a dox-inducible shRNA (top left) targets the Ndufa4l2 region of the LucR-Ndufa4l2 fusion transcript, thereby decreasing the expression of LucR protein. The LucR activity was normalized to the activity of LucF expressed from a constitutive active promoter (HSV-TK). Dox-inducible shRNA expression was confirmed by visualization of EGFP in more than 95% of the stably transfected cells. **(C)** Generation of a stable cell line and the mating scheme for breeding the triple-transgenic TANdu mouse line. ES cells were nucleofected with plasmid constructs cTGME (targeting vector) and pCAGs-Flpe (expression vector) for the targeted integration into the recipient FRT-site in the *Col1a1* locus. Correct integration introduces expression of the HygR selection marker, and is confirmed using Col1A1(+), Col1A1 (–), and SAdpA (–) primers (red, green, and blue triangles, respectively). Validated ES cells were injected into blastocysts to generate shNdufa4l2 transgenic animals. Three validated shNdufa4l2 males were mated with females carrying the transgenic HIF1α (TRACK) and a ggt-driven tet-Transactivator (ggt tTA). **(D)** Ndufa4l2 protein levels are decreased in the shNdufa4l2 mice (shNdu) relative to the TRACK ccRCC model (to 57 ± 7%). Kidney cortex protein was isolated from kidneys of three male TANdu mice (6 months old, ID#19-259). In addition, protein was isolated from age-matched male WT and TRACK mice (one and three mice, respectively). The antibodies used are specified in the Method section.

We next developed a strategy for generating a genetically engineered mouse model (GEMM) in which a ggt-driven shRNA can effectively reduce Ndufa4l2 transcript levels specifically in the proximal tubules (**Figure 2**). We designed several shRNA hairpins for Ndufa4l2-targeting using Splash and transOMIC online resources (**Table S4**). The Ndufa4l2-targeted shRNAs were inserted into the dox-inducible L3GGEPPIR vector, as well as the cTGME targeting vector (**Figure 2A**). Notably, the cTGME construct facilitates targeting into the FRT-Hygro modified *Col1a1* locus (20). We also generated a LucR/LucF dual luciferase reporter construct in which the LucR coding region was expressed in cis to the Ndufa4l2 cDNA (NM_001098789.1). The LucF expression cassette in this construct provided a reference for normalization.

We stably transduced F9 teratocarcinoma cells to generate polyclonal cell lines for each shRNA sequence. We then transiently transfected the luciferase reporter construct into polyclonal F9 cell lines (each harboring a specific shRNA for Ndufa4l2), and the following day treated the cells with either vehicle or doxycycline to induce shRNA expression. The subsequent depletion of the LucR-Ndufa4l2 transcript was inferred by measuring the LucR activity relative to the activity of the LucF reference gene. By this approach we quantified the targeting efficiencies of individual shRNAs, which ranged from 15 ± 4% (shNdufa4l2.39) to 79 ± 1% (shNdufa4l2.304) depletion (**Figure 2B**). Based on this assessment, shNdufa4l2.304 was selected for efficient genomic targeting.

We introduced the cTGME targeting vector into the FRT-Hygro modified *Col1a1* locus of HK2 mouse ES cells as previously described (20), and used qualitative PCR for genotyping of derived mouse strains (**Figure 2C**). We cross-bred the resulting mouse lines to generate a triple transgenic line which harbors the TRACK transgene (activated HIF1a), a tet-Transactivator (ggt-tTA) driving tTA expression in the PT cells, and the tet-regulated shNdufa4l2.304. In this novel mouse line, which we named TANdu (tTA-shNdufa4l2), endogenous Ndufa4l2 levels are negatively regulated by PT-specific expression of the Nduf4l2-targeting shRNA. In this context, the shRNA is co-expressed with GFP under control of a ggt-driven tet-transactivator (tTA), and addition of dox will inhibit shRNA expression. We utilized this triple transgenic mouse model to delineate the actions of Ndufa4l2 in the TRACK model of early ccRCC (11).

### The Spatial Distribution of GFP Marks shRNA Expression and the Local Depletion of Ndufa4l2 Protein

We evaluated expression of the shRNA (GFP) and the target (Ndufa4l2) in kidney cortices of three TANdu mice and three TRACK mice (**Figure 2D**). A normal kidney cortex (WT) served as negative control. We detected comparable levels of hairpin expression (GFP) in the TANdu samples. Importantly, we found that Ndufa4l2 protein levels were reduced in the TANdu mice by 57 ± 7% (p=0.03) relative to levels in the TRACK parental line (**Figure 2D**). Thus, findings confirmed a selective knockdown of Ndufa4l2 protein levels in TANdu kidneys.

**Figure 3:**
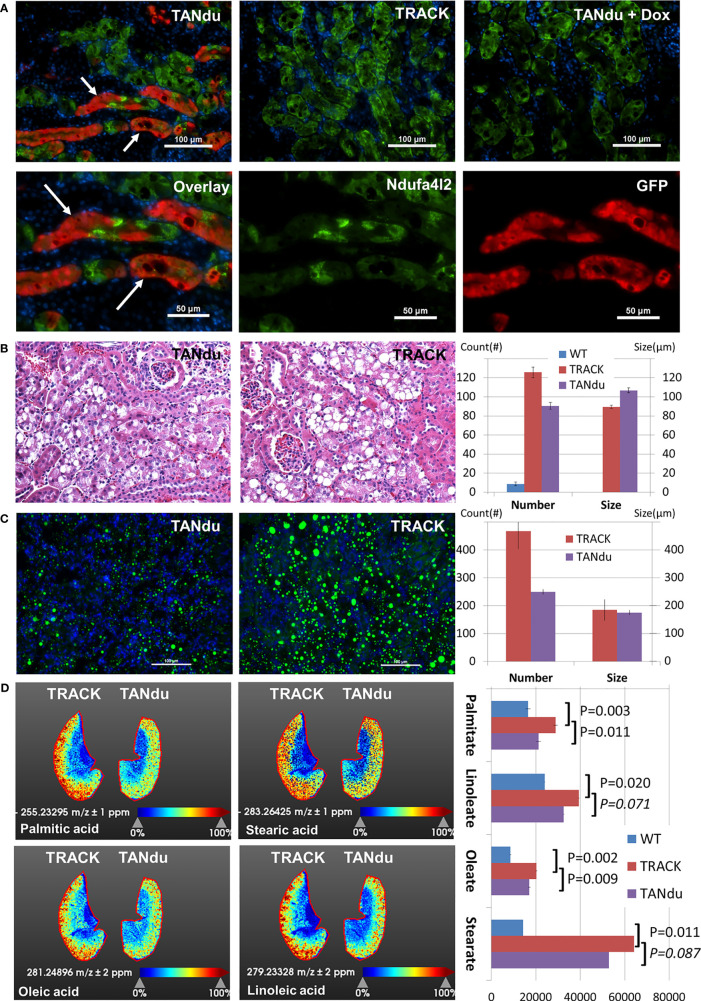
Biological Effects of Ndufa4l2 depletion. **(A)** Immunofluorescence of Ndufa4l2 (green), GFP (red), and cell nuclei (blue). Marked by white arrows; Ndufa4l2 (visualized by green staining) is reduced specifically in the proximal tubules, which express the shRNA (stained in red). The patchy Ndufa4l2 staining (green) is unlike that of TRACK where the staining extends throughout the entire area of each individual tubule. In the top panels are shown TANdu; no doxycycline supplementation. TANdu+Dox; doxycycline in the drinking water (one month duration immediately prior to sacking). TRACK; the parental line. The staining in TRACK marks the areas in which the HIF1a transgene is expressed (i.e. the proximal tubules). Note how the staining extends throughout the entire area of each individual tubule (versus the patchy staining in the TANdu). Part of the TANdu section is enlarged in the lower panels showing overlay (left) and individual channels of Ndufa4l2 and GFP (center and right, respectively). Magnification; x200, scale bar; 100 μm (50 μm in split channels). Representative images are shown. The antibodies used are listed in **Table S5**. WT kidneys are negative for Ndufa4l2 staining (see **Figure 1**). The mice were 6 months old, ID#19-376. **(B)** Histology of TANdu and TRACK kidney cortices. The H&E stain indicates comparable lipid deposition in TANdu and TRACK kidneys. Automatic quantification in ImageJ identifies lower numbers of lipid droplets (of a larger average size) in TANdu relative to TRACK images (number of droplets per field of view). **(C)** Spatial Distribution of Neutral Lipids. The Bodipy stain of frozen kidney sections identifies decreased abundance of lipid droplets in TANdu kidneys relative to those of TRACK mice. Kidney sections were stained from three mice of each genotype. **(D)** Spatial Distribution of Fatty Acids. Heatmaps of TANdu and TRACK kidneys. Heatmaps indicate the relative levels of Palmitate, Stearate, Oleate, and Linoleate as determined by MALDI-IMS. Note that these fatty acids accumulate predominantly in the kidney cortices. Kidney sections were imaged from two mice of each genotype, including WT mice. Quantification of Palmitate, Stearate, Oleate, and Linoleate as determined by MALDI-IMS in WT, TRACK and TANdu kidneys. Each of the four free fatty acids are significantly elevated in TRACK (relative to WT), whereas Palmitate and Oleate are significantly decreased in TANdu (relative to TRACK).

The decreases in Ndufa4l2 levels in TANdu kidney cortices could reflect moderate depletion in most or all PTs, or major reductions in a subset of Ndufa4l2 expressing tubules. To distinguish between these possibilities we immunostained mouse kidneys for GFP, which functions as a proxy for the expression of Ndufa4l2-targeting shRNA. Additionally, we counterstained with Ndufa4l2 to determine if shRNA expression effectively decreased the level of its target protein (**Figure 3A**). Results showed that GFP expression followed a tubular pattern in the TANdu kidneys, but also noted a “patchy” penetrance of the GFP expression (**Figure 3A**). Importantly, tubular regions with more intense GFP staining displayed less intense endogenous Ndufa4l2 protein (see white arrows), thus demonstrating the expected inverse relationship between Ndufa4l2 shRNA expression (marked by GFP) and endogenous Ndufa4l2 protein levels (**Figure 3A**, the left hand panels show overlay of the three channels). The overlap between the Ndufa4l2 and GFP positive protein stains was only 3.6% ( ± 0.6%) of the total area of Ndufa4l2 (67 ± 5%) and GFP (29 ± 4%) staining per field (**Figure S2**), indicating that when the hairpin is expressed (GFP), the Ndufa4l2 protein is efficiently depleted. For comparison, the colocalization of Ndufa4l2 and Eno1 protein stains showed a 72% overlap (**Figure S2**). The TRACK parental line, which does not express the Ndufa4l2-shRNA transgene (**Figure 3A**, top center), served as negative control. To address potential leakiness of the hairpin, we evaluated testis, pancreas, and liver of adult TANdu males for GFP expression (**Figure S3**), and detected GFP expression only in the kidney cortex. To determine the dox-responsiveness of the shRNA, GFP expression was evaluated in the TANdu line following one month of dox treatment (**Figure 3A**, top right). As evident by the lack of GFP staining, the dox treatment prevented the ggt-driven tTA protein from inducing the shRNA transgene. This result confirmed that by supplementing the drinking water with dox, the *in vivo* shRNA expression can be turned off pharmacologically. We conclude that in the TANdu mouse model Ndufa4l2 expression can be selectively attenuated in a reversible manner for a subset of PTs, presumably the PTs in the S1 segment where the Ggt1 promoter is known to be active (21). In order to further ascertain that the hairpin is expressed specifically in the proximal tubules of the TANdu kidneys, we performed immunostaining for proximal tubule markers Lotus Tetragonolobus Lectin, Ggt1, Sglt1, and Aqp1 (21, 31–33) and superimposed the staining with that of GFP (**Figure S4**). For each of the four markers we found that a subset of cells were also positive for GFP, demonstrating that the hairpin for targeting Ndufa4l2 is expressed in a subset of proximal tubule cells. Furthermore, staining the basement membrane and brush borders with wheat germ agglutinin (34), we found that the GFP expression stained individual tubules (**Figure S4**). Lastly, expression of E-Cadherin, a marker of collecting ducts, did not show any overlap with GFP expression (**Figure S4**).

### The TANdu Kidneys Exhibit Lower Lipid Content

To assess the effects of Ndufa4l2-depletion we first evaluated the general morphology of H&E-stained TANdu and TRACK kidneys (**Figure 3B**). A pathologist (F.K.) blinded to the section identities noted only minor differences in the overall “clear cell” areas in the TANdu kidneys relative to those of TRACK. In order to further refine this observation we used image analysis tools (Fiji/ImageJ) to determine the number and sizes of lipid droplets in each field of view (FoV) for multiple images collected from at least three mice of each group (WT, TRACK, and TANdu, **Figure 3B**). We found that the average number of lipid droplets was decreased in the TANdu kidneys relative to TRACK (90 ± 3.7 vs 126 ± 5.5 per FoV, p=0.026). Conversely, the average size of the lipid droplets increased in the TANdu kidneys relative to TRACK (107 ± 2.6 vs 89 ± 1.6 per FoV, p=0.004).

In the H&E tissue stains, “clear cell” areas were identified structurally (as the de-paraffinized sections are stripped of lipids). In order to directly detect renal lipids, we stained frozen sections with the fluorescent Bodipy dye, which specifically recognizes neutral lipids (**Figure 3C**). Consistently, we found decreased numbers of neutral lipid droplets in the TANdu kidneys relative to those of TRACK (249 ± 37 vs 467 ± 63 per FoV, p=0.023). The average size of the lipid droplets was unchanged in the TANdu kidneys relative to TRACK (176 ± 8.5 vs 184 ± 9.0 per FoV, p=0.745).

To identify differences in lipid composition between TANdu and TRACK kidneys, we mapped the spatial distributions of specific fatty acids using MALDI-IMS (**Figure 3D**). We found significantly decreased levels of both palmitate (16:0) and oleate (18:1), whereas decreases in the levels of stearate (18:0) and linoleate (18:2) did not reach statistical significance (imaging results of WT kidneys and biological replicates of TANdu and TRACK kidneys are depicted in **Figure S5**). The decreased abundances of these long chain fatty acids and in overall neutral lipid content is in accord with the possibility that decreased Ndufa4l2 levels may accelerate fatty acid β-oxidation. We also noted increased levels of hexose-phosphate in TRACK relative to WT kidneys (190337 ± 42417 vs 113888 ± 15537), suggesting increased glycolytic activity in the TRACK kidneys (**Figure S6**). Interestingly, TANdu displayed decreased hexose-phosphate (175563 ± 32583) relative to TRACK kidneys (decreased by 8%, p=0.02). Finally, we noted decreased levels of AMP, ADP, and ATP in TRACK relative to WT kidneys, but depletion of Ndufa4l2 did not reverse these levels. Renal lactate levels did not change among WT, TRACK, and TANdu mice (**Figure S6**).

### The ccRCC Markers Car9 and Eno1 Are Reduced in Ndufa4l2 Depleted Tubules

**Figure 4:**
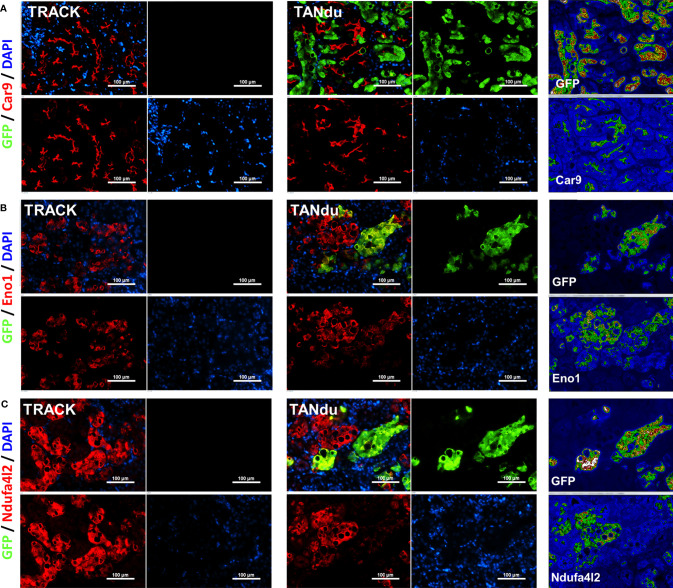
Expression of Car9 and Eno1 is reduced in proximal tubules expressing shNdufa4l2. Immunofluorescence of Car9 (red, **(A)**, Eno1 (red, **(B)**, Ndufa4l2 (red, **(C)**, GFP (green, marking the expression of shNdufa4l2), and cell nuclei (blue). Staining of TRACK kidneys is shown to the left, and heat-maps of staining intensities in the TANdu kidneys is shown to the right. Panels show representative immunostainings of kidney cortices from three independent mice of each genotype. Magnification; x200, scale bar; 100 μm. Representative images are shown. The antibodies used are listed in **Table S4**.

To assess additional effects of Ndufa4l2-depletion, we next stained kidney cortices from three TANdu mice for the ccRCC biomarkers Car9 and Eno1 (red), and concomitantly counterstained with GFP (green) to identify tubules positive for Ndufa4l2-shRNA expression (**Figure 4**). We observed the characteristic cup-like staining pattern for Car9 in both TANdu kidneys and in TRACK kidneys (**Figure 4A**). Notably, Car9 staining was markedly less pronounced in regions that displayed intense GFP staining (see heatmap in **Figure 4A**).

**Figure 5:**
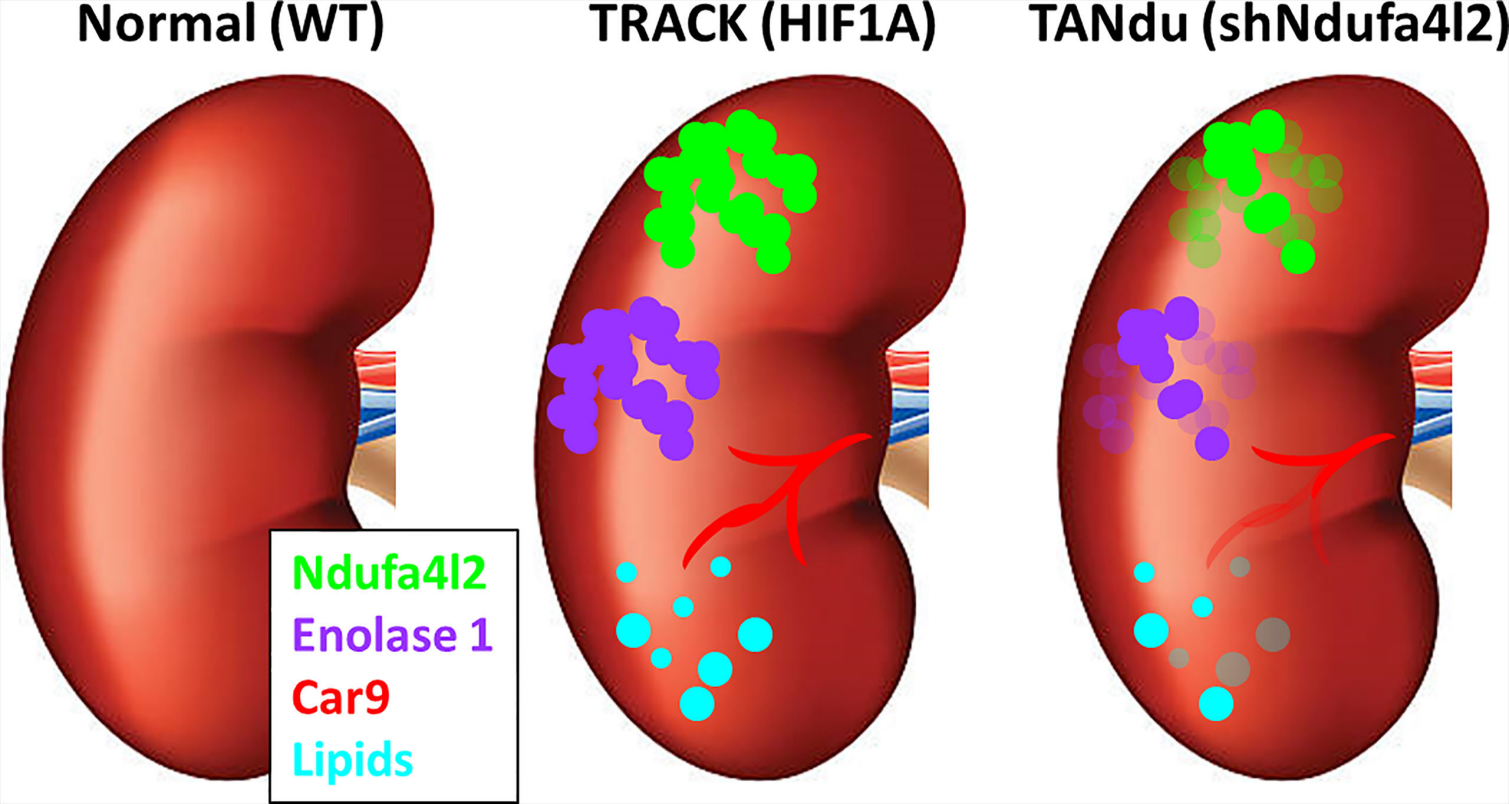
Summary of the effects of Ndufa4l2. In contrast to normal kidneys, TRACK kidneys exhibit lipid accumulation (cyan), and are positive for Ndufa4l2 (green), Enolase1 (purple), and Car9 (red). In the TANdu kidneys, the decreased level of Ndufa4l2 protein (relative to TRACK kidneys) is accompanied by decreased levels of Eno1 and Car9, and reduced lipid accumulation, suggesting that decreasing the levels of Ndufa4l2 lessens the ccRCC pathologies.

We observed the characteristic tubular staining patterns for Eno1 (**Figure 4B**) and Ndufa4l2 (**Figure 4C**) in both TANdu and TRACK kidneys. Furthermore, tubular cells with more intense GFP staining displayed less intense Eno1 staining (see heatmap in **Figure 4B**), indicating that decreasing Ndufa4l2 protein levels is associated with a demonstrable reduction in Eno1 expression levels. Our finding that ccRCC protein biomarkers Car9 and Eno1 decrease in abundance when expression of Ndufa4l2 is attenuated suggests that high Ndufa4l2 expression may be a critical driver of the lipid accumulation and high CA9 levels associated with ccRCC. Our findings are graphically summarized in **Figure 5**.

## Discussion

Perturbations in mitochondrial metabolism have emerged as a key player in cancer progression (35–37). We have previously demonstrated that constitutive HIF1A-activation in kidney PTs induces neoplastic transformation leading to the development of precancerous lesions that closely resemble ccRCC (11). These lesions are typified by increased transcription of a number of glycolytic enzymes and metabolite transporters (10, 11). In contrast, transcription of only a few mitochondrial proteins results from HIF1A-activation. One of the most profound increases is that of Ndufa4l2, a mitochondrial protein shown to be required for proliferation and colony formation in kidney cancer cell lines (10, 15). Importantly, Ndufa4l2 is also elevated in human ccRCC (15, 38), and this high expression in cancer makes NDUFA4L2 a promising target for therapeutic approaches. In the present study, we decreased Ndufa4l2 expression in our TRACK ccRCC animal model to evaluate the effects of targeting Ndufa4l2. We found that depletion of Ndufa4l2 was associated with decreased levels of Car9, the mouse homolog of the commonly used ccRCC marker CA9. We also discovered that a reduction in Ndufa4l2 decreased the level of Eno1, a key glycolytic enzyme implicated in c-Myc signaling (39, 40). Finally, we demonstrated that Ndufa4l2 expression can be restored by providing the mice with doxycycline-supplemented drinking water, thus providing a dynamic approach for reversible depletion of Ndufa4l2 in an animal model of early ccRCC.

Transcription of both Car9 and Eno1 is induced by HIF1A-activation (41, 42), and consequently, both Car9 and Eno1 levels are elevated in ccRCC (29, 43). In addition to the direct regulation by HIF1A, the transcriptional activation of CA9 depends on the metabolic state of the cell, e.g. CA9 transcription is enhanced by the presence of lactate (44), and ENO1 responds to cellular c-Myc activity (45). In addition to its role in glycolysis, ENO1 is involved in cell growth, hypoxia tolerance, and autoimmune activities (46), in part by binding directly to the c-Myc promoter to repress transcription of c-Myc (40). We observed decreases in both CA9 and ENO1 protein levels in Ndufa4l2-depleted tubule cells, and since Ndufa4l2 resides in the mitochondrial inner membrane (14) we speculate that these decreased CA9 and ENO1 protein levels result from altered cellular metabolism. Human ccRCC is characterized by elevated levels of pyruvate and lactate, products of increased glycolysis. We previously showed that these metabolites are also elevated in our TRACK model (4). Endogenous Ndufa4l2 has been reported to inhibit mitochondrial respiration, so depletion of Ndufa4l2 is anticipated to enhance oxidative phosphorylation. We noted decreased levels of hexose-phosphate in the TANdu kidneys (relative to the TRACK kidneys), suggesting that Ndufa4l2 levels could modulate the rate of glycolysis, possible by regulating oxidative phosphorylation. An increase in mitochondrial respiration would predictably divert pyruvate from lactate production into the TCA cycle, and the resulting decrease in lactate accumulation would likely have a negative impact on CA9 transcription (44). We did not, however detect any significant changes in lactate among WT, TRACK, and TANdu. It is interesting to note that lactate was recently reported to be incorporated directly into the histone tails, where it facilitates enhanced transcriptional activity (47).

Fatty acid synthesis is linked to mitochondrial function. Specifically, hypoxic activation introduces reductive carboxylation of glutamine, which in turn produces Acetyl-CoA for fatty acid synthesis (48). Depletion of Ndufa4l2 may shift the TCA cycle from reductive carboxylation to oxidative decarboxylation, thus decreasing the amount of Acetyl-CoA available for *de novo* fatty acid synthesis.

In summary, we show that the well-established histological features of ccRCC, including lipid deposition and elevated expression of key cancer biomarkers (i.e., CA9 and ENO1), are significantly decreased with Ndufa4l2 knockdown in our TANdu mouse model. These findings suggest that targeting NDUFA4L2, either alone or potentially in combination with mTor and tyrosine kinase inhibitors, offers a novel potential path for effective therapies in ccRCC patients.

## Data Availability Statement

The original contributions presented in the study are included in the article/**Supplementary Material**. Further inquiries can be directed to the corresponding author.

## Ethics Statement

The animal study was reviewed and approved by WCMC Institutional Animal Care and Use Committee.

## Author Contributions

KL, LD, and LG: conception and design. LG, DN, and SG: administrative support. KL, QC, FK, and NA: collection and assembly of data. KL and QC: data analysis and interpretation. All authors contributed to the article and approved the submitted version.

## Funding

Funded in part by The Weiss Family, NIDDK-R01, DK113088 (LJG), and Weill Cornell Funds, R01AR076029, R21 ES032347, and R21 NS118233 (SSG, QC).

## Conflict of Interest

The authors declare that the research was conducted in the absence of any commercial or financial relationships that could be construed as a potential conflict of interest.

## Publisher’s Note

All claims expressed in this article are solely those of the authors and do not necessarily represent those of their affiliated organizations, or those of the publisher, the editors and the reviewers. Any product that may be evaluated in this article, or claim that may be made by its manufacturer, is not guaranteed or endorsed by the publisher.

## Abbreviations

ETC, electron transport chain; GEMM, genetically engineered mouse model, ccRCC, Clear cell renal cell carcinoma; oxphos, oxidative phosphorylation; VHL, von Hippel-Lindau tumor suppressor; tTA, tet-Transactivator; LucF, Luciferase, Firefly; LucR, Luciferase, Renilla; ggt, gamma-glutamyltransferase 1 promoter; PT, proximal tubules; dox, Doxycycline; TRACK, Transgenic Cancer of the Kidney; FoV, field of view.
