# Supplementary material for: Mitochondrial Ndufa4l2 Enhances Deposition of Lipids and Expression of Ca9 in the TRACK Model of Early Clear Cell Renal Cell Carcinoma
Source: Front Oncol. 2021 Dec 14;11:783856. doi: 10.3389/fonc.2021.783856 (PMC8712948; doi:10.3389/fonc.2021.783856)
Supplement: Supplementary file 1 [file DataSheet_1.pdf]

Supplementary Figures and Tables

Figure S1. Ndufa4l2 expression in kidney, testis, pancreas, and liver of adult TRACK males. The different tissues were processed in parallel, and noticeable Ndufa4l2 expression (green) was found only in the kidney. DAPI nuclear staining (blue). Magnification; x200, scale bar; 100 µm. Images were acquired from at least three independent animals. Representative images are shown.

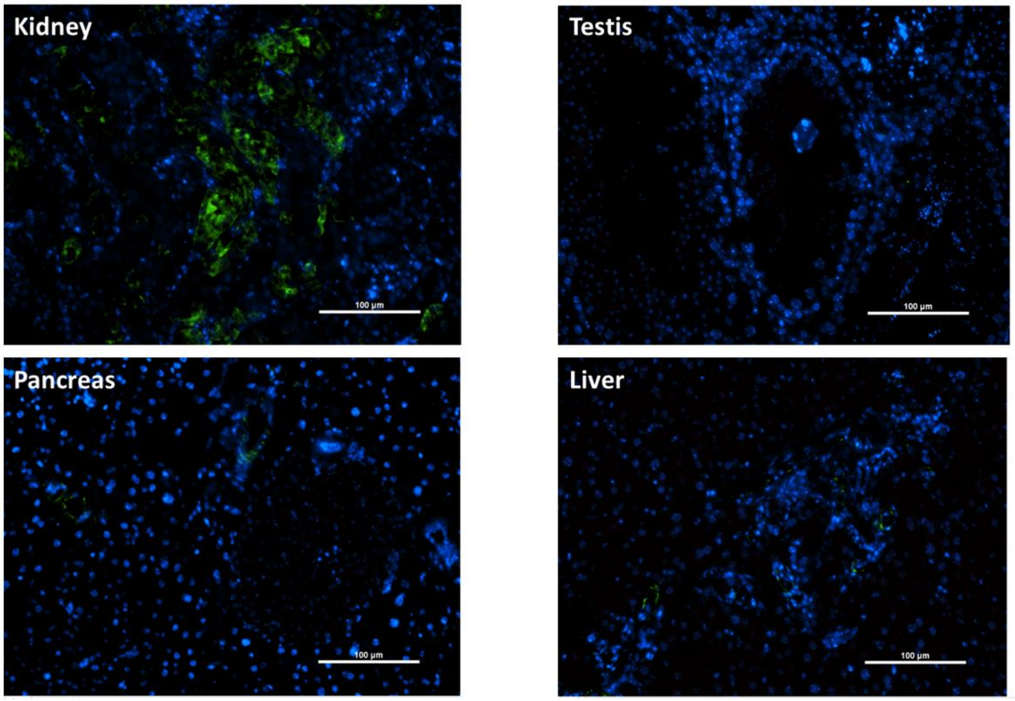

Figure S2. Quantification of spatial overlap between Ndufa4l2 and GFP stainings. Images of kidney cortices from five TANdu mice provided the input data for ImageJ analysis. The staining intensities of Ndufa4l2 (green) and GFP (red) were gated by automated thresholding, and the positive areas were quantified (Ndufa4l2; 67±5%, GFP; 29±4%). The overlay between the two areas was measured relative to the total area stained (3.6±0.6%). As a positive control for overlapping staining, the overlap between Ndufa4l2 and Enolase1 was determined using the same algorithm (72%). Magnification; x200, scale bar; 100 µm.

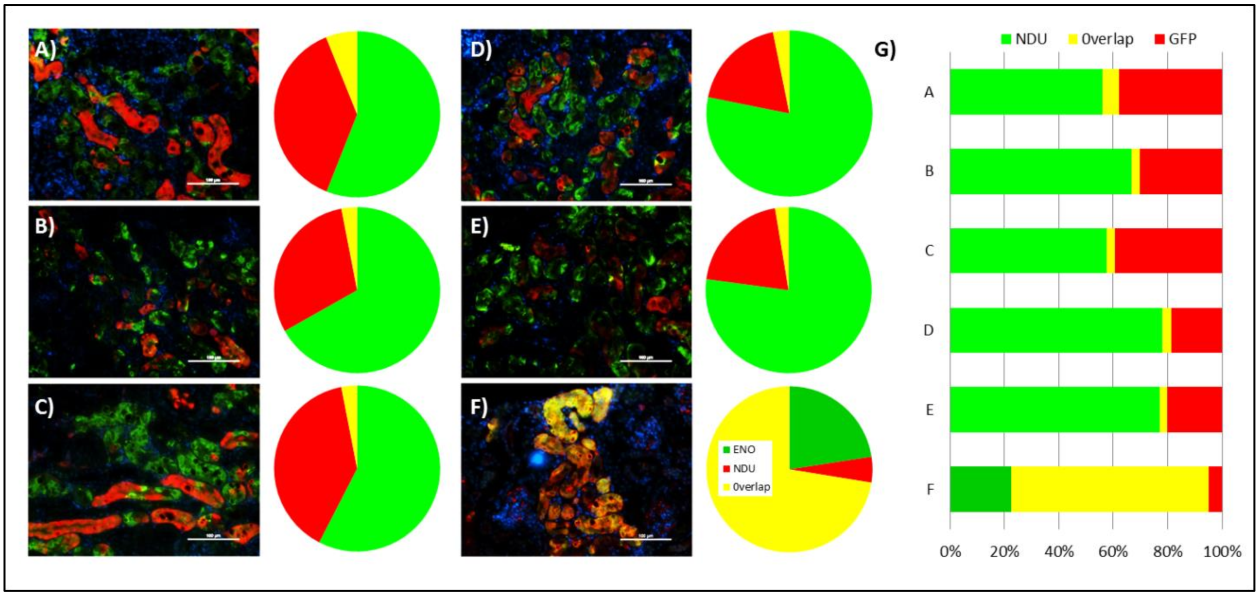

Figure S3. Transgene expression in kidney, testis, pancreas, and liver of adult TANdu males. The different tissues were processed in parallel, and noticeable GFP transgene expression (red) was found only in the kidney. Wheat germ agglutinin staining (WGA, green) provides structural information. DAPI nuclear staining (blue). Magnification; x200, scale bar; 100 µm. Images were acquired from at least three independent animals. Representative images are shown.

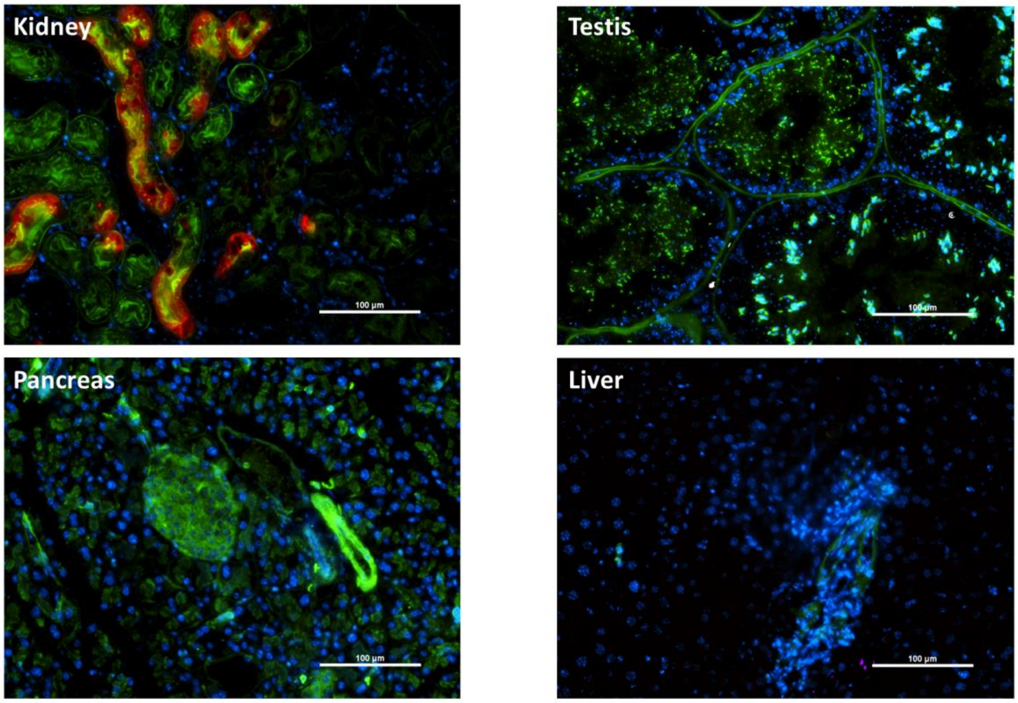

Figure S4. Spatial overlap between stainings for GFP and markers of the proximal tubules. Expression of GFP (shown in red) is restricted to a subset of proximal tubule cells positive for *Lotus Tetragonolobus* Lectin (LTL), gamma-glutamyltransferase-1 (Ggt1), Sodium/glucose cotransporter-1 (Sglt1), or Aquaporin-1 (Aqp1), all shown in green. Staining with Wheat germ agglutinin (WGA) and E-Cadherin (both in green) are shown for reference. Magnification; x200, scale bar; 100 µm. Images were acquired from at least three independent animals. Representative images are shown.

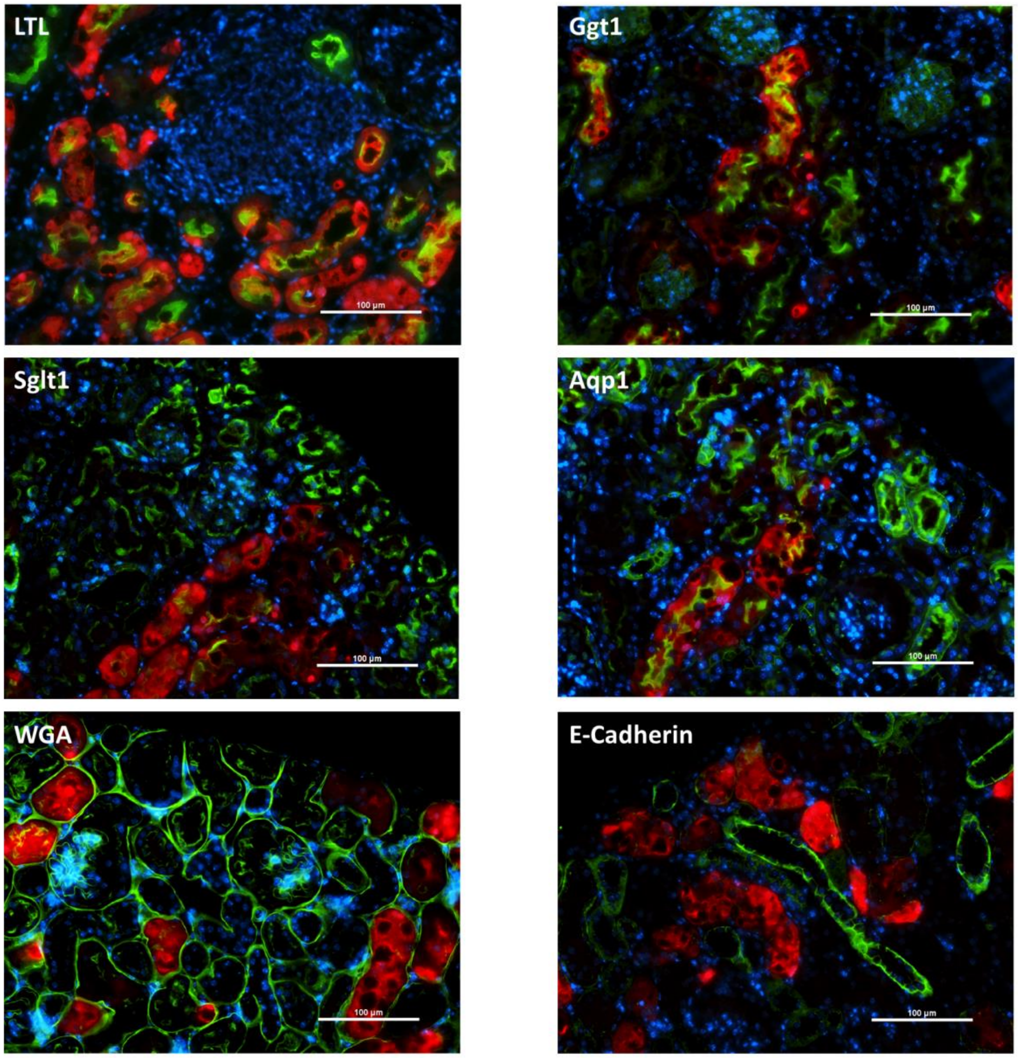

Figure S5. Spatial Distribution of Fatty Acids depicted as heatmaps of WT, TANdu and TRACK kidneys. Heatmaps indicate the relative levels of Palmitate, Stearate, Oleate, and Linoleate as determined by MALDI-IMS.

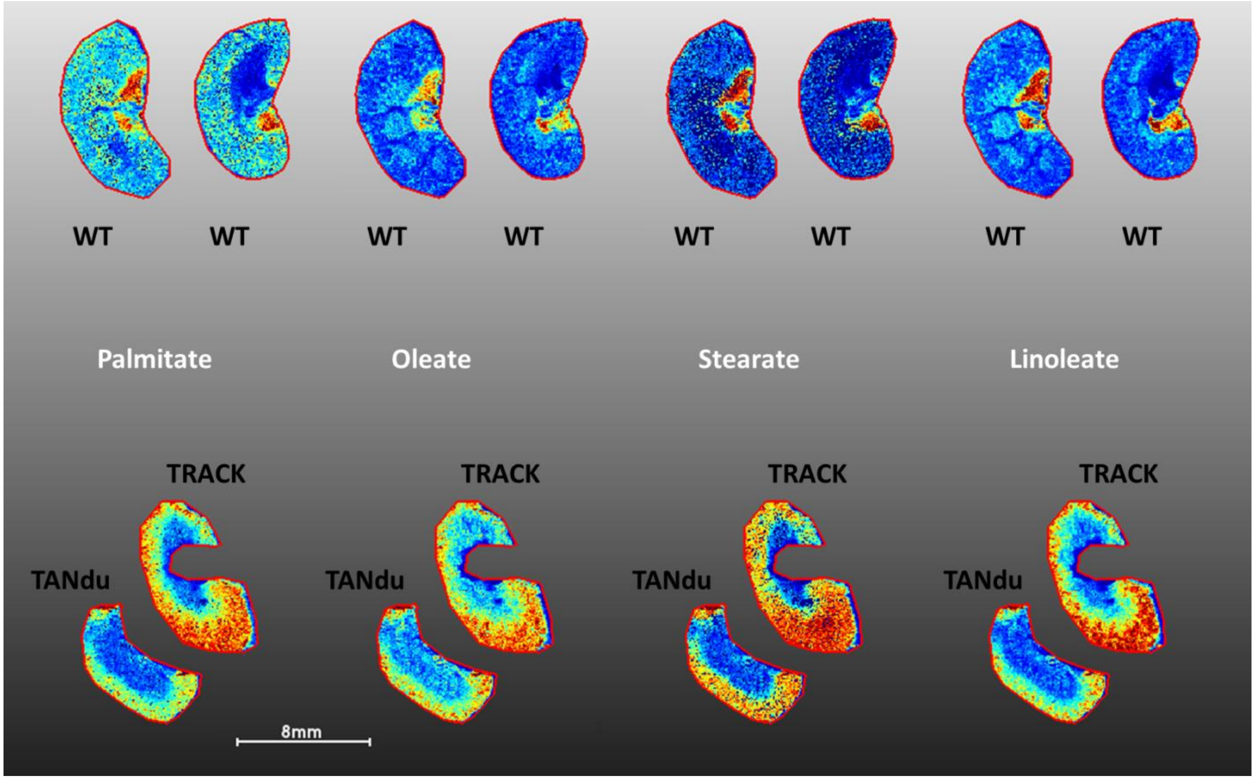

Figure S6. Spatial Distribution of select metabolites depicted as heatmaps of WT, TANdu and TRACK kidneys. Heatmaps indicate the relative levels of A) Hexose-phosphate, B) AMP, C) ADP, D) ATP, and E) Lactate as determined by MALDI-IMS.

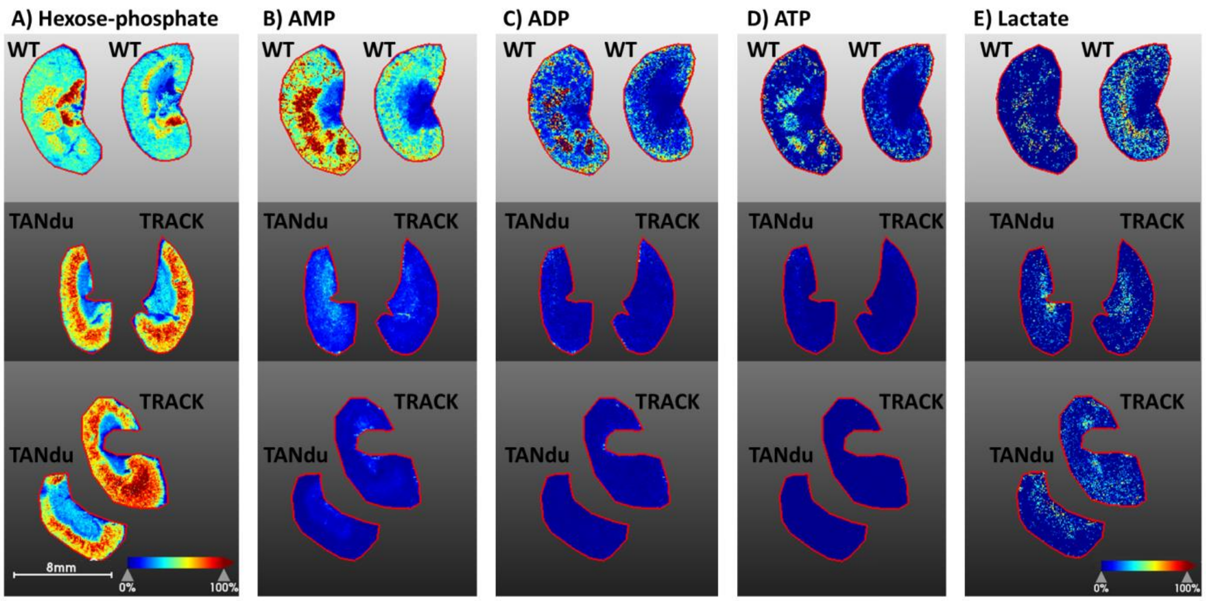

Supplementary Tables

Primer	Sequence
miRE-Eco-Rev	TCTCGAATTCTAGCCCCTTGAAGTCCGAGGCAGTAGGC
miRE-Xho-Fw	TGAACTCGAGAAGGTATAT
mNdufa4l2(-)E4	AAACGGCCGAATTTGTTAAATGAAATTGTATTTTGCCCA
mNdufa4l2(+)X1	ATCGCTCGAGAAGACCAGACACTGGGACAAGAT

Table S1. Construction Primers. The miRE PCR products were cloned into the L3GGEPPIR and cTGME vectors. The Ndufa4l2 PCR product was cloned into psiCHECK2.

Primer	Sequence
EGFP(-)	CGCCGGACACGCTGAACTTGTG
TRE3G(+)A	ACCATGCATGTGTTCGATTCT
TETO(+)B	GAGCTCGGTACCCGGGTCG
tta507_F	GCTGCTTAATGAGGTCGG
tta507_R	CTCTGCACCTTGGTGATC
Col1A1(+)	AATCATCCCAGGTGCACAGCATTGCGG
Col1A1(-)	ACTTGAGGGCTCATGAACCTCCCAGG
SAdpA(-)	ATCAAGGAAACCCTGGACTACTGCG
hHIF1aP564A-F	TTGGAGATGTTAGCTGCCTATATCCC
hHIF1aN803A-R	AACTTCACAATCATAACTGGTCAGC

Table S2. Genotyping Primers.

Forward	Reverse	gDNA	Description
Col1A1(+)	Col1A1(-)	220	Col1a1-WT
Col1A1(+)	SAdpA(-)	295	Col1a1-Integration
tta507_F	tta507_R	507	GGT-tTA
TRE3G(+)A	EGFP(-)	526	TRE3G (BPS)
TETO(+)B	EGFP(-)	261	TetO (shRNA)
hHIF1aP564A-F	hHIF1aN803A-R	732	TRACK

Table S3. PCR products for genotyping.

Primer	Sequence
Adaptors	TGCTGTTGACAGTGAGCG------------shRNA------------TGCCTACTGCCTCGGA
NDUFA4L2.1152 #355	ACGTGGGCAAAATACAATTTCATAGTGAAGCCACAGATGTATGAAATTGTATTTTGCCCACGG
NDUFA4L2.1157 #361	AGCAAAATACAATTTCATTTAATAGTGAAGCCACAGATGTATTAAATGAAATTGTATTTTGCC
NDUFA4L2.477 #sh3	ACAGTTTCCACTGACTATAAGATAGTGAAGCCACAGATGTATCTTATAGTCAGTGGAAACTGC
NDUFA4L2.463 #349	CATACAAGTTCCTTGCAGTTTATAGTGAAGCCACAGATGTATAAACTGCAAGGAACTTGTATT
NDUFA4L2.478 #sh5	AAGTTTCCACTGACTATAAGAATAGTGAAGCCACAGATGTATTCTTATAGTCAGTGGAAACTG
NDUFA4L2.460 #sh6	CCCAATACAAGTTCCTTGCAGTTAGTGAAGCCACAGATGTAACTGCAAGGAACTTGTATTGGT
NDUFA4L2.406 #367	CCTGCTGGGACAGAAAGAACAATAGTGAAGCCACAGATGTATTGTTCTTTCTGTCCCAGCAGA
NDUFA4L2.472 #sh8	CCCTTGCAGTTTCCACTGACTATAGTGAAGCCACAGATGTATAGTCAGTGGAAACTGCAAGGA
NDUFA4L2.266 #352	ACGACTACAAGAAGCTGAAGAATAGTGAAGCCACAGATGTATTCTTCAGCTTCTTGTAGTCGG
NDUFA4L2.243 #359	ATACAAGTTCCTTGCCGTTTCATAGTGAAGCCACAGATGTATGAAACGGCAAGGAACTTGTAC
NDUFA4L2.244 #360	CACAAGTTCCTTGCCGTTTCAATAGTGAAGCCACAGATGTATTGAAACGGCAAGGAACTTGTA
NDUFA4L2.39 #353	AAAGATGGCAGGAACTAGTCTATAGTGAAGCCACAGATGTATAGACTAGTTCCTGCCATCTTG
NDUFA4L2.304 #354	CAAGTCAGAGATCTCCACGTAATAGTGAAGCCACAGATGTATTACGTGGAGATCTCTGACTTA
NDUFA4L2.291 #350	ACGGCCAGACTTCTAAGTCAGATAGTGAAGCCACAGATGTATCTGACTTAGAAGTCTGGCCGG
NDUFA4L2.289 #356	AACCGGCCAGACTTCTAAGTCATAGTGAAGCCACAGATGTATGACTTAGAAGTCTGGCCGGTC
NDUFA4L2.316 #357	ATCCACGTAAATCACTCTTTCTTAGTGAAGCCACAGATGTAAGAAAGAGTGATTTACGTGGAG
NDUFA4L2.239 #358	CCCAGTACAAGTTCCTTGCCGTTAGTGAAGCCACAGATGTAACGGCAAGGAACTTGTACTGGT
NDUFA4L2.406 #369	CCTGCTGGGACAGAAAGAACAATAGTGAAGCCACAGATGTATTGTTCTTTCTGTCCCAGCAGA
NDUFA4L2.472 #370	CCCTTGCAGTTTCCACTGACTATAGTGAAGCCACAGATGTATAGTCAGTGGAAACTGCAAGGA
NDUFA4L2.1157 #513	AGCAAAATACAATTTCATTTAATAGTGAAGCCACAGATGTATTAAATGAAATTGTATTTTGCC

Table S4. Hairpin sequences for Ndufa4l2 knockdown. The hairpin-sequences were PCR amplified with primers miRE-Eco-Rev_oligo and miRE-Xho-Fw_oligo (Table S1) and cloned into L3GGEPPIR and cTGME vectors (Figure 2).

Target	WB	IF	Catalog#	Lot#	Company	Specie
**Ndufa4l2**	1:1000	1:200	16480-1	00007801	ProteinTech	rabbit
**Eno1**	1:1000	1:200	11204-1-AP	00014144	ProteinTech	rabbit
**GFP**	1:1000	1:200	STJ140006	000666270214	St.John's Lab	goat
**Car9**	NA	1:200	50660-R001	HA07MC0806	Sino Biological	rabbit
**Gpi1**	1:1000	1:200	15171-1-AP	00007145	ProteinTech	rabbit
**Ggt1**	NA	1:200	A1776	0006510201	AbClonal	rabbit
**Sglt1**	NA	1:200	PA5-88282	WC3216903	Thermo	rabbit
**Aqp1**	NA	1:200	MA5-32593	WB3190355	Thermo	rabbit
**E-Cad**	NA	1:200	3195S	13	Cell Signaling	rabbit
**LTL**	NA	1:1000	FL-1321	ZF-0801	Vector	n/a
**WGA**	NA	1:1000	L-4895	ND	Sigma	n/a
**rabbit**	NA	1:500	A32790	TI271740	Thermo	donkey
**goat**	NA	1:500	A32758	TI271731	Thermo	donkey
**actin**	1:5000	NA	MAB1501	2951837	Millipore	mouse
**rabbit**	1:5000	NA	711-035-152	110017	JacksonLabs	donkey
**mouse**	1:5000	NA	715-036-150	108896	JacksonLabs	donkey

Table S5. Anti-bodies used in the study.
